# Adaptive Node Clustering for Underwater Sensor Networks

**DOI:** 10.3390/s21134514

**Published:** 2021-06-30

**Authors:** Muhammad Fahad Khan, Muqaddas Bibi, Farhan Aadil, Jong-Weon Lee

**Affiliations:** 1 Department of Computer Science, Attock Campus, COMSATS University Islamabad, Attock 43600, Pakistan; m.fahad@cuiatk.edu.pk (M.F.K.); muqaddasbibi74@gmail.com (M.B.); 2 Department of Software, Sejong University, Seoul 05006, Korea

**Keywords:** underwater sensor networks, nodes clustering, dragonfly optimization, optimized routing, transmission range, adaptive node clustering technique, ANC-UWSNs

## Abstract

Monitoring of an underwater environment and communication is essential for many applications, such as sea habitat monitoring, offshore investigation and mineral exploration, but due to underwater current, low bandwidth, high water pressure, propagation delay and error probability, underwater communication is challenging. In this paper, we proposed a sensor node clustering technique for UWSNs named as adaptive node clustering technique (ANC-UWSNs). It uses a dragonfly optimization (DFO) algorithm for selecting ideal measure of clusters needed for routing. The DFO algorithm is inspired by the swarming behavior of dragons. The proposed methodology correlates with other algorithms, for example the ant colony optimizer (ACO), comprehensive learning particle swarm optimizer (CLPSO), gray wolf optimizer (GWO) and moth flame optimizer (MFO). Grid size, transmission range and nodes density are used in a performance matrix, which varies during simulation. Results show that DFO outperform the other algorithms. It produces a higher optimized number of clusters as compared to other algorithms and hence optimizes overall routing and increases the life span of a network.

## 1. Introduction

A large part of Earth is covered with oceans [1]. Oceans have large populations of animals and other creatures. They are the main source of food, transport and other resources for many creatures on earth. With the progression of society and economy, increasingly individuals have begun to focus on marine conditions. Marine condition frameworks are especially presented with the impacts of human activities linked to industry and urban advancement tourism [2,3]. In the early days of oceanic exploration, human beings interests in this research were very limited due to harsh environment of oceans e.g., large areas and high water pressure, oceanic research unmanned investigation. In 20th century wireless sensor networks become very popular in research area. Initially WSNs was only used in terrestrial environments. With the passage of time, new progression in the technology of the oceanic modem made wireless sensor networks applicable in underwater environments [4]. In this environment, deployment and management of WSNs is more difficult as compared to land-based environments. Monitoring of an underwater environment and communication is essential for many applications. The scope of these applications ranges from observing climate, underwater environment, marine ecology, natural calamities, navigation, and transmission of images to remote side on land, offshore investigation, surveillance and other military applications [5,6,7,8,9].

Wireless Sensor Networks (WSNs) have been reflected as feasibly favorable alternates for observing oceanic environs due to their easy positioning, immediate monitoring, and fairly low cost. Underwater sensor networks consist of two sections, sensor nodes and data collecting stations known as sink. Sensor nodes can track and sense physical parameters and send data to a base station using wireless communication technologies [10]. Generally, radio frequency waves are most commonly used for communication in wireless sensor networks (WSN), but due to high signal attenuation and the short distance range of radio frequency waves, these are not recommended to use in underwater sensor network for communication. There is need of designing new routing protocols for underwater communication. Various approaches for underwater communication are proposed for example, underwater acoustic communication (UAC), underwater optical communication (UOC), electromagnetic and magnetic field communication. Other than UAC and UOC, all techniques are on research laboratory authentication phase. Drawback of optical communication is scattering of signals. So UAC method is most commonly used now days. Oceanic signals range is up to few kilo meters in underwater environment [11]. As compared to terrestrial environment, water environment is very complex and changeable, so ocean monitoring is very difficult. Underwater sensor network (UWSN) is Ad-Hoc network responsible for underwater communication. Underwater sensor network contain nodes with marine on board modems and limited amount of surface stations. Sensor nodes can send and receive data between source and destination. Sensor nodes perform three main tasks, monitor data, process data and receive data. These nodes are of different types to sense and monitor different physical or chemical parameters in underwater environments and send the collected data to the central location.

Early routing protocols were designed for specific layers e.g., protocols designed for network layer or transport layer. These protocols tailed layered protocol architecture. They did not use joint optimization, energy levels and due to some other causes overall performance is meager, so current research focus to design protocols which can use data received by different layers called cross layered routing protocols.

Considering the above issues, there are some other problems which make underwater communication challenging. One such issue is limited bandwidth. It directly affects the transmission rate of a network. Water current and different sources of noise result in limited bandwidth in UWSNs [11]. Propagation delay is another issue which cannot be neglected for efficient routing. Propagation delay of oceanic signals is very high and is 2 × 10^5^ time high than the propagation delay in WSNs [5]. For long distance communication in underwater sensor networks, oceanic waves are used which can travel thousands of kilometers at low frequency and high power [11]. Underwater network faces a high bit error rate and loss of connectivity due to multipath interference of oceanic channels [5]. Due to the multipath effect, noise, water temperature and Doppler spread underwater communication is unreliable. The multipath effect also fade the coming signals. Due to these problems, routing has become challenging and costly in UWSNs [12].

**Figure 1:**
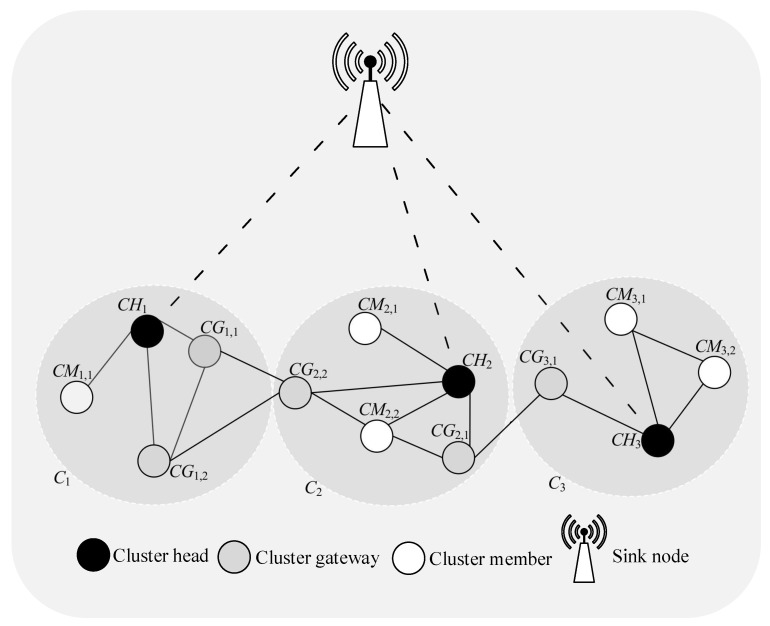
Node clustering architecture for underwater sensor networks (UWSNs).

To address the above listed problems, an effective routing protocol must be created. The proposed technique will split UWSN into clusters and provide dynamic cluster head selection system in Figure 1. Clustering means, rally of sensor nodes into logical sets. These nodes sense data and send to the base station. One node is chosen as cluster head and remaining nodes in the relay are called member nodes. All inter- and intra-communication of cluster is done by cluster head. Cluster head selection will depend upon remaining energy of nodes. When cluster head energy is less than a certain value then a new CH will be selected [13]. The proposed protocol based on the dragon fly optimization algorithm (DFO). Dragon fly optimizer is an evolutionary based optimizer which creates optimized number of clusters and hence reduces the number of hops, so life span of the network increases.

The main contributions of this research are as follows:

- An evolutionary algorithm will be used for optimization of UWSN’s routing. An evolutionary algorithm is based on the social behavior of many different insects and animals. These algorithms provide optimized routing solutions. They are successfully used in MANET (mobile area network), VANET (vehicle area network) and FANET (flying area network) [14]. In This paper dragon fly optimization (DFO) technique is used for routing optimization to make UWSN more efficient.
- The results of this paper will be equated with other evolutionary algorithms and outcomes and illustrate that proposed paper shows batter results.

The remaining paper will be divided in following segments. Segment 2 represent related work, segment 3 represent the main notions of dragon fly optimization (DFO), segment 4 discusses proposed methodology, and segment 5 has details of performance evaluation, results, and discussion.

## 2. Related Work

This section is categorized into *five* main parts are as follows.

### 2.1. DBR Routing Technique

Many protocols designed for UWSNs communication to route data on correct destination efficiently. One technique used for energy efficient routing is depth-based routing (DBR). In this technique, the depth of sensor node is measured, using this evidence data from lower nodes sent to receiver nodes. Depth measurement is done by a depth sensor fixed inside the node. Depth and residual energy information is used for energy efficient deep routing, but end to end delay is high in DBR. Energy efficient DBR (EEDBR) is another protocol designed for an underwater environment. It has two phases, one is the formation acquirement phase and other is data forwarding phase. In initial phase sensor node selects its neighbor node on the bases of more remaining energy and low depth from base node and save (Id) in the routing table. In the second stage, with the use of their routing tables, each node forward data packs to the next node which has high remaining energy and least sink depth. Optimization Depth based routing (ODBR) [15] system guarantees consistency in consumption of energy between sensor nodes to maximize life span of network. Reza Mohammedi proposed a new fuzzy depth based routing (FDR) approach [16]. In this approach, for routing decisions, Fuzzy cost based on a receiver’s residual energy, depth difference between previous forwarded node and receiver node and number of hops passed by received packet. It achieves better results than the novel DBR in average end-to-end delay, packet transmission rate and energy saving. Another depth-based protocol named as enhanced energy efficient (EEEDBR) proposed for UWSNs [17]. It chooses the node with high remaining energy and low depth from sink for data forwarding. In a middle depth area, idle nodes are installed to overcome extra transmission of data packets and hence increase the life time of network. In this scheme, nodes are also capable to change the transmission range to a certain limit. CDBR [18] is another scheme in which cluster heads (CH) used to forward data packs and increase the efficiency of network. It outperforms as compared to EEDBR, which is a light-weight depth-based routing protocol (LWDBR) [19] based on a residual energy method for minimizing energy ingestion.

### 2.2. Clustering Technique

Clustering is a famous technique for saving energy in sensor networks [20]. One of the clustering-based routing protocols is (MCCP) minimum cost clustering routing protocol for (UWSNs). It includes three main parameters: the first is the total amount of energy used by member nodes while transmitting data to cluster head, the second is total remaining energy of cluster head node and its member nodes, and the last parameter is locality among cluster head and base station. When a cluster is formed then the cost of each cluster is calculated using the above three parameters. A re-clustering mechanism is done also to maintain traffic load and energy of nodes, but end to end delay is very high in this technique. Another technique known as distributed underwater clustering technique (DUCS). In (DUCS) with the help of distributed algorithm clusters are made. Nodes shape themselves into confined cluster; each cluster chooses a node as a single cluster head. All member nodes transfer data to its cluster head through a single hop. This protocol reduces proactive routing communication and uses data aggregation to eradicate redundant material. Another clustering technique used in UWSNs is (EBECRP) energy efficient and balanced energy consumption cluster based routing protocol [21]. In this technique, mobile sinks are used to maintain the load of overall nodes and clustering of nodes reduces multi hop selection and energy consumption. This method is energy efficient and performance effective. Cluster based energy efficient routing protocol (CBE2R) [22] designed for underwater environment. In this approach oceanic depth is divided into seven layers. On these layers courier powerful nodes embedded from top to bottom to increase the battery power of nodes. Courier nodes gather information from sensor nodes and sent to base node. These courier nodes are also called cluster head and have more energy and memory as compared to sensor nodes. Courier nodes sends “join” message to neighbors nodes to form cluster. Fuzzy based energy efficient routing protocol in [23] proposes a fuzzy based cluster head selection model to improve the life span of network. Another clustering approach (CMSE2R) [24], the proposed protocol uses cluster based multipath shortest distance energy efficient routing for UWSNs. CMSE2R divided into four stages first stage is network setting, second stage is cluster creation, third stage is multipath growth in the related clusters and last stage is transfer of data. This protocol increases the link quality among nodes by using static carrier nodes and developing a multipath in related clustering techniques. It gives better results as compared to (CBE2R).

### 2.3. Vector-Based Forwarding Technique

A vector-based forwarding technique is also used in underwater sensor networks for improving routing. A vector-based forwarding routing protocol (VBF) was proposed by Peng Xie. This is a location-based approach specially designed to improve packet delay and data transmission rate. Data are sent from source to destination by those nodes which are near to “vector” so that only few nodes contribute in data forwarding. The drawback of this protocol is that end to end delay is very high. Double-Sink Vector Based Forwarding (DSVBF) protocol incorporates both remaining energy and location data as a primary factor in finding optimized routes to save energy in underwater networks. The use of dual sink increases the life span of an underwater sensor network. In fuzz logic vector-based forwarding routing protocol (FVBF) [25], node location and energy information is used in the calculation. Some other parameter also measured which are valid distance of nodes and battery power of nodes. It shows good results than (VBF) and (LEVBF). Another vector-based routing protocol proposed in [26]. This protocol improves the (VBF) technique which depends upon the radius of routing pipe. This paper presented an algorithm that treats the pipe radius as a function of the surrounding dimension as well as the range and amount of nodes. Similarly, there is a function for energy ingestion control. If the energy of a receiver node is much inferior to the source node, this method shrinks routing pipeline radius to reduce the probability, that this node is used as guiding node. So, other nodes may have the opportunity to obtain the data packet of leading node. With this new protocol named Directional Vector Forward Focused Beam Routing (DVFBR) [27], UWSN routing will be made more dynamic.

### 2.4. LEACH Algorithm

The Low Energy Adaptive Cluster Head Selection (LEACH) [28] algorithm is most commonly used in WSNs and also in UWSNs. The main function of this algorithm is to reduce energy consumption of sensor networks. The article [29] raised a system having low energy clustering hierarchy, designed for underwater sensor networks. When a member node selects a cluster to join, it consider the position of the cluster head and energy ingestion between member nodes and cluster head, also energy consumption between cluster head and sink. UMOD-LEACH [30] protocol is modification of Leach protocol and modified algorithm shows 30% better results than (LEACH) in underwater environment. It uses localization concept and time division multiple access (TDMA). Another adaptive clustering routing system (ACUN) was proposed for underwater networks in [31]. It uses multipath routing based on two main parameters, remaining energy of (CH) node and distance among (CH) and sink. Leach-MAC [32] is also the modification of this algorithm which works more efficiently in WSNs. Similarly, region-based (R-Leach) and modified weighted (W-Leach) proposed in [33,34].

### 2.5. Intelligent Algorithms (PSO, GWO, MFO)

Some other intelligent algorithms are mostly used for efficient data routing in WSNs, e.g., particle swarm optimizer (PSO), gray wolf optimizer (GWO) and moth flame optimizer (MFO). Particle Swarm Optimization (PSO) based technology is widely used in many optimization calculation problems. Many protocols designed for WSNs by using (PSO) technique. One of them is an FSO-PSO clustering-based technique proposed for WSNs in [35]. In this scheme, two optimizers are used, fish swarm optimizer and particle swarm optimizer and perform efficient multi hop clustering. This hybrid technique improves life time and scalability of network. Another scheme is “low energy PSO based node positioning” [36] designed for WSNs. This scheme uses PSO to optimize nodes position with the lowest consumed energy. By optimizing nodes location, nodes energy consumption is effectually condensed, and whole performance of system is enhanced. In [37], mobility prediction based particle swarm optimization (MPPSO) technique proposed for UWSNs in which position and speed of node is calculated through this technique. It decreases the positioning period of mobile node and energy ingestion. Fuzzy and PSO-based clustering techniques were proposed for UWSNs by Vani Krishnaswamy [38] for efficient data routing. There are four main steps, (1) cluster making by fuzzy technique, (2) counting of clusters considering error factor, (3) fitness function formulation including following parameters (consumed energy, distance among cluster nodes and from sink),(4) CH selection using ‘PSO’. It shows better results than LEACH algorithm.

#### 2.5.1. GWO

A gray wolf optimization algorithm (GWO) is also used in many WSNs for optimization of clustering. In (GWO-LPWSN) [39], the proposed protocol uses gray wolf optimization technique for positioning of nodes and for improving the errors in positioning. It shows better results than PSO. In [40] the proposed technique provide a transmission rate control mechanism based on (GFO) for WSNs. Support vector machine is used for controlling transmission rate of nodes and for tuning of (SVM) gray wolf optimizer is used. In [41], the protocol has three levels for clustering optimization, in first level sink node selects the cluster head, in second level nodes selects best route for data transmission by using GFO, in the last level clustering function is formed. Similarly, in [42], gray wolf optimization and genetic algorithms are used for optimized clustering.

#### 2.5.2. MFO

A moth flame optimizer is another intelligent algorithm used to solve optimizations problems. MFO is motivated by the natural behavior of moths and provides optimized solutions for NP-hard problems. A protocol designed in [43] is based on the MFO algorithm for optimized clustering. Similarly in [44] moth flame and genetic algorithm used for optimized and energy aware clustering. Due to its batter results it is also used in UWSNs. (SOSNET) [45] is another routing protocol designed for smart oceans. It uses moth flame optimizer (MFO) which performs optimized clustering in cost effective way. Similarly, in [46], the proposed technique solves the problem of fault tolerant routing using MFO which selects the best route for data transmission.

## 3. Dragonfly Algorithm

**Figure 2:**
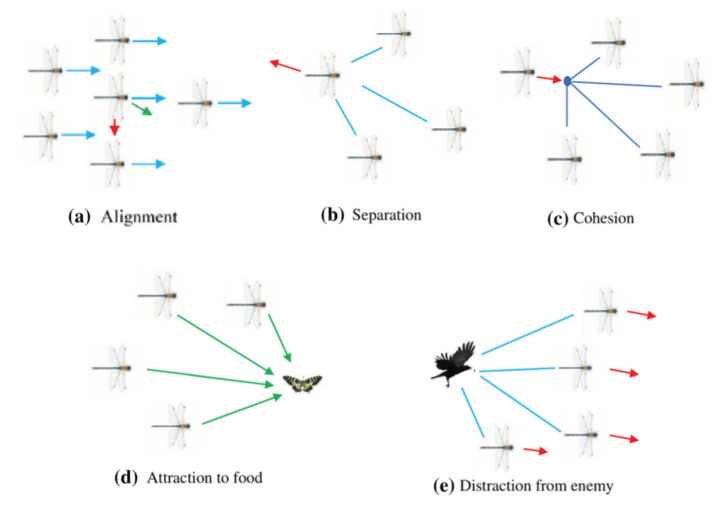
Primitive corrective patterns between dragonflies in a swarm (different steps of the artificial dragonfly algorithm).

Dragonflies are elegant insects, having almost 3000 different species around the world. In the life cycle of a dragonfly, adults lay eggs in water and from eggs they become nymphs, spend most of the time of their life in this stage and then by metamorphism they become adults. Dragonflies have individual and rare swarming techniques. They have two main swarming techniques; static (feeding) and dynamic (migratory) swarm. Dragons make small clusters for food hunting in static swarm while in dynamic swarm large groups of dragons migrate over long distance [47]. This algorithm used in many optimization problems in WSNs. In [48] a protocol designed for VANET environment use DFA algorithm for optimized selection of cluster heads efficiently. Similarly in [49] also DFA algorithm used for optimization. The basic motivation of DFA starts with static and dynamic swarming behavior of dragonflies. These swarming behaviors are similar to optimization stages: exploration and exploitation [48,50]. Exploration and exploitation based on *five* principles as shown in Figure 2.

- *Separation*: individuals avoid collusion from other localities.
- *Alignment*: individual’s speed correspondence with other localities.
- *Cohesion*: individual’s tendency towards the central mass of neighborhoods.
- *Attraction towards source*: individuals attracted towards food source.
- *Distraction from enemies*: individuals distracted from enemies.

Above mentioned factors are utilized for position upgrading of individuals. Equations for these factors are given below.

(1) $$S=-\sum_{j=1}^{N}\left(P-P_{j}\right)$$

In Equation (1), *S* shows separation, *P* shows current individual position and $P_{j}$ shows *j*th neighboring individual position and *N* shows total individuals.

(2) $$A=\sum_{j=1}^{N}\left(S_{j}/N\right)$$

where $S_{j}$ is velocity of *j*th individual in Equation (2).

(3) $$C=[\sum_{j=1}^{N}\left(P_{j}/N\right)]-P$$

(4) $$E_{n}=P^{-}+P$$

(5) $$F_{o}=P^{+}-P$$

where $P^{-}$ and $P^{+}$ are the positions of enemy and food respectively and *P* is position of current individual in Equations (4) and (5).

For artificial dragonflies, two main vectors will be used to update their position, step vector (ΔP) and position vector (*P*). The framework of DFO depends on PSO working and step vector works same like velocity vector in PSO. Step vector and position vector calculated by given equations respectively.

(6) $$P_{T+1}=P_{T}+\Delta P_{T+1}$$

(7) $$\Delta P_{T+1}=\left(sS_{i}+aA_{i}+cC_{i}+eE_{n_{i}}+fF_{o_{i}}\right)+w\Delta P_{T}$$

In Equation (7), ($S_{i},\;A_{i},\;C_{i},\;En_{i},\;Fo_{(}i)$) shows separation, alignment, cohesion, distraction from enemy and attraction towards food of *i*th individual and (s, a, c, e, f) are weights respectively, *w* is the weight step vector at time (*T*), *T* shows the current iteration. In the enhancement process, altered exploratory and exploitative practices completed for optimization.

DFO is a population-based optimizer, where dragons display sensor nodes in UWSNs and with the help of dragon position, a matrix of MN can be created; M shows dragon’s number and N shows total variables. An additional array is formed to hold dragon fitness values known as dragon array. This fitness value is calculated by the fitness function for each dragon. Dragons are the agents move in the search space and in the each iteration, position vector provide best position to these dragons. In this way, a DFO algorithm provides the best optimal solution in a heuristic way.

**Figure 3:**
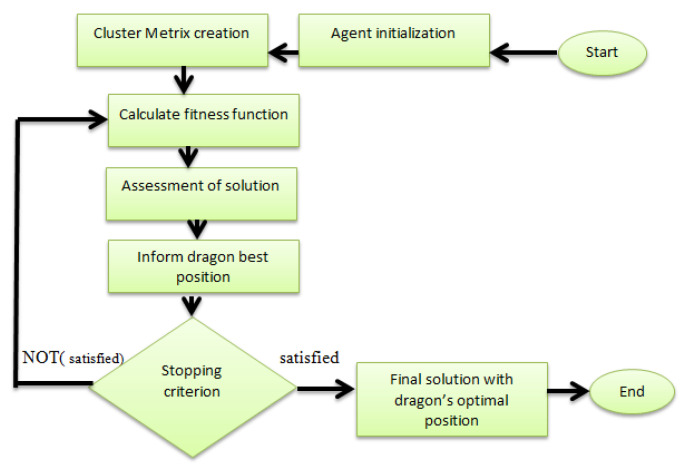
Flowchart of dragonfly (DF) algorithm.

A flow diagram of DFO is shown in Figure 3. In the initialization time, dragonflies in the MN dimensional space have an arbitrary position and fitness values of each dragonfly stored in the dragon array. Correspondingly position matrix and position array is also created. The best value of dragons is stored in the position matrix so far. The dragonflies move in the search space until the solution reaches its optimal state and then search operation is completed. At the end dragonfly’s best position is updated. A similar procedure is used repetitively, till optimum location for each dragonfly is attained.

## 4. Proposed Methodology

In [46], a DFO algorithm is used in a clustering-based optimization technique designed for a VANET environment. In the proposed paper, we use the same algorithm for underwater sensor networks (UWSNs). Due to the evolutionary capabilities of this solution and good result in a VANET environment, we use this algorithm in UWSNs for finding optimal number of clusters. We have created M×N grid of sensors in a geographical region. In a Mesh network, all sensor nodes are provided with appropriate IDs and these sensor nodes are considered as dragons. A search space is created on the basis of these dragonflies. Distance between all nodes is calculated by euclidean distance and stored in a matrix. The search space based on dimensions, lower bond and upper bound parameters. Fitness of all dragonflies is calculated by using their positions in the search space. Fitness values of all dragonflies are stored in the fitness matrix. As this process is iterative so in each iteration calculated values of fitness stored in matrix and this matrix give dragonflies lower fitness values. By combining dragon fitness and position values, a best score is calculated and on the basis of this score, the positions of dragonflies are updated. So this converge to optimal solution by using a decreasing factor and we get optimum clusters necessary to communicate effectively depending on assumed parameters. The algorithm of proposed protocol is shown in Figure 3. After cluster formation, the next phase is CH selection. For this purpose, the following parameters are used; density of nodes, transmission range of nodes, grid size. All these parameters were assigned weights in fitness function. A fitness function is calculated to find the best possible solution from all the candidates solutions. A fitness function is very significant in the algorithm. By selecting the right cluster head will improve the life span of cluster and potentially contribute to network energy saving. In the proposed algorithm, a fitness value is calculated by the following equation.

(8) $$Fitness\;function=w_{1}\times a_{1}+w_{2}\times a_{2}$$

where $a_{1}$ and $a_{2}$ depicts $delta_{difference}$ and $distance_{neighbor}$, respectively,

In Equation (8), $delta_{difference}$ is the difference and $distance_{neighbor}$ is the average distance of nodes. $delta_{difference}$ is used as criterion for load balance. $w_{1}$ is the weight allocated to $delta_{difference}$ and $w_{2}$ is the weight allocated to $distance_{neighbor}$. In some situations, all clusters will have equal numbers of nodes, but in a real world scenario, it is not easy due to sensor nodes position changes because of water current and some other influences. $delta_{difference}$ is used for measuring the variance from an ideal degree to movement of a node from its neighbors. It is calculated as;

(9) $$delta_{difference}=abs\left(ideal_{deg}-node_{deg}\right)$$

Recent work has shown that, due to static CH selection criteria, there is a high probability that one parameter may skew the fitness function and improper CH selection may occur, so in the proposed solution, weights are allotted to all parameters dynamically, on the basis of negative influence on fitness function, reliant on situation. In this process, the first value of each parameter isnormalized between 0 to 10 and then deviation of each parameter is calculated by the equation:

(10) Dev(p)=[mean−parameter(p)]

The total amount of all weights shall be approximately equal to 1. Each node’s fitness value is determined by using Equation (8), where values of parameters are used and weights assigned to each parameter in the equation.

## 5. Performance Evaluation, Results and Discussion

### 5.1. DFO Based Algorithm for UWSNs

The details for the DFO based Algorithm 1 for UWSNs and its algorithm have been discussed in Section 4.
**Algorithm 1.** Dragon Fly Optimization based algorithm for UWSNs
1:**procedure**2:    Install all nodes in 2D net randomly3:    Set the direction of each node randomly4:    Set the velocity of each node5:    Create MESH topology between all nodes6:    Calculate the distance between nodes and associate these values to edges7:    Create search space and initialize dragonflies8:    Initialize $C_{min}$ and $C_{max}$9:    Compute fitness of early swarm10:       **for** iterations = 1 to stall iteration (stall iteration is set to 10)12:           **while** (Nodes! = empty)13:             Nodes clustering = All Node14:          **end while**15:           **while** I ≤ iterations16:             **for** 1 to Population size = 100 (Dragonflies)17:              Standardize distances Amid Search agents18:              Update Location of current Search Agent19:              Bring all Agents with in Upper Bound (UB) and lower bound (LB)20:            **end for**18:             Update Best cost19:             Iteration+120:           **end while**21:         Best cost22:       **end for**20:**end procedure**

### 5.2. Experimental Setup

The experiment was performed using the 2018a edition of MATLAB testing performed on 7th generation, corei5 system with 16 GB RAM. Much experimentation was performed utilizing altered grid size varied from 500 m to 2000 m. Similarly, node density ranging from 20 to 200 is used in the tests and nodes transmission range also change ranging from 25 m to 200 m. Nodes are supposed to stay in fixed locations or move very slowly due to water current. DFO was contrasted with other state of the art evolutionary clustering protocols, which are ant colony optimizer (ACO), comprehensive learning particle swarm optimizer (CLPSO), gray wolf optimizer (GWO) and moth flame optimizer (MFO). All the parameters for simulation are shown in Table 1.

### 5.3. Simulation Results and Discussions

**Figure 4:**
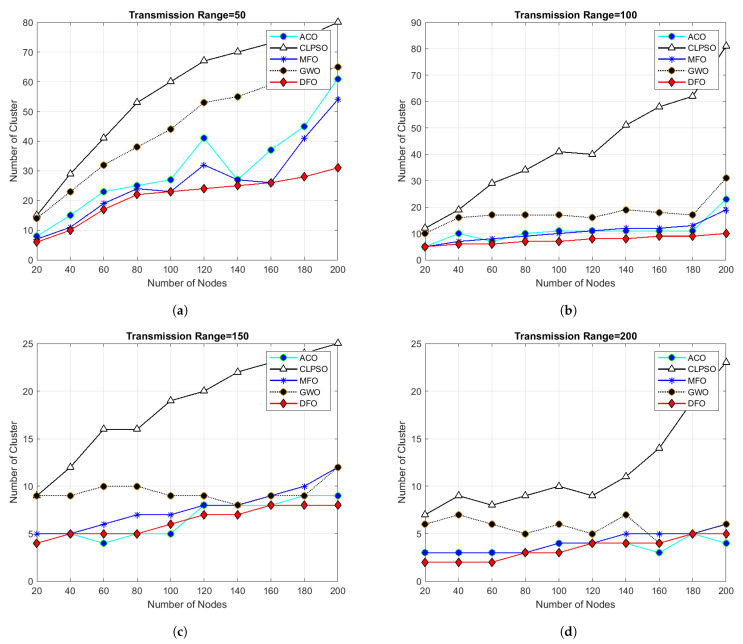
Grid size 500 m × 500 m and number of nodes 20 to 200.

**Figure 5:**
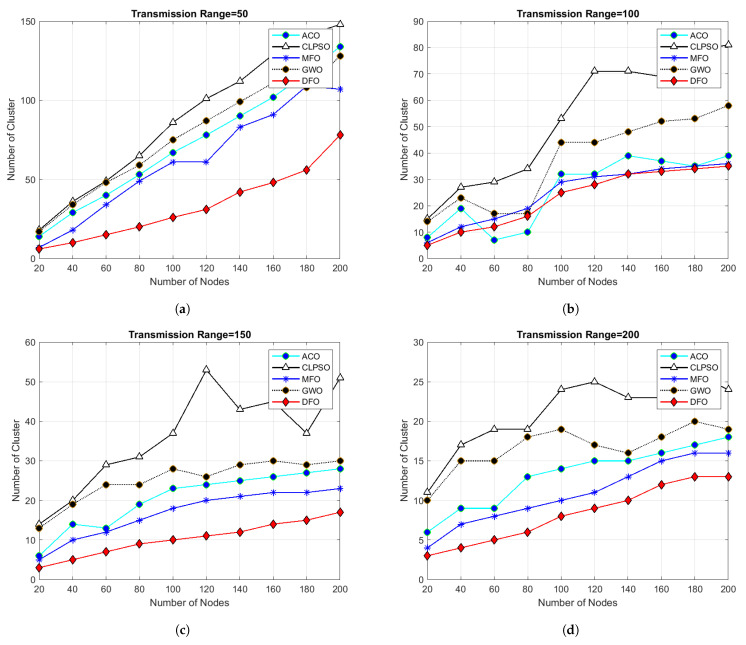
Grid size 1000 m × 1000 m and number of nodes 20 to 200.

**Figure 6:**
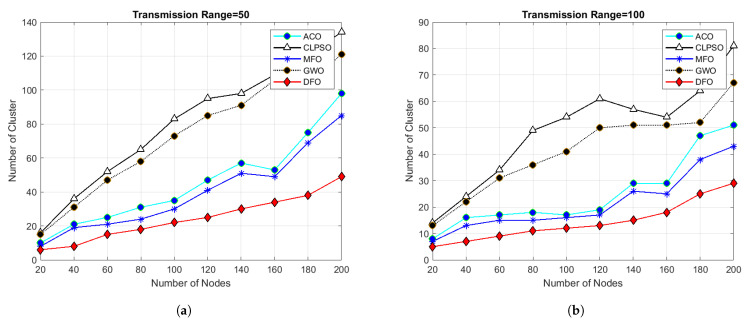

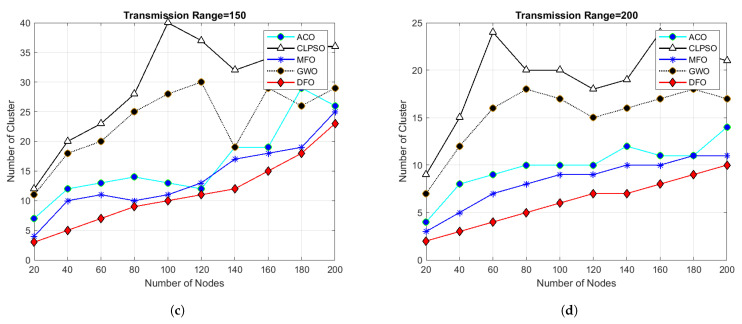
Grid size 1500 m × 1500 m and number of nodes 20 to 200.

**Figure 7:**
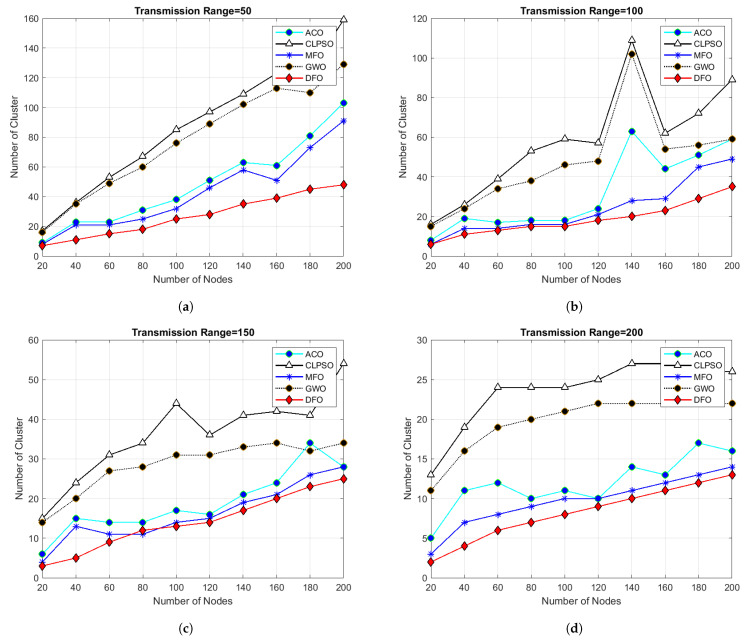
Grid size 2000 m × 2000 m and number of nodes 20 to 200.

**Figure 8:**
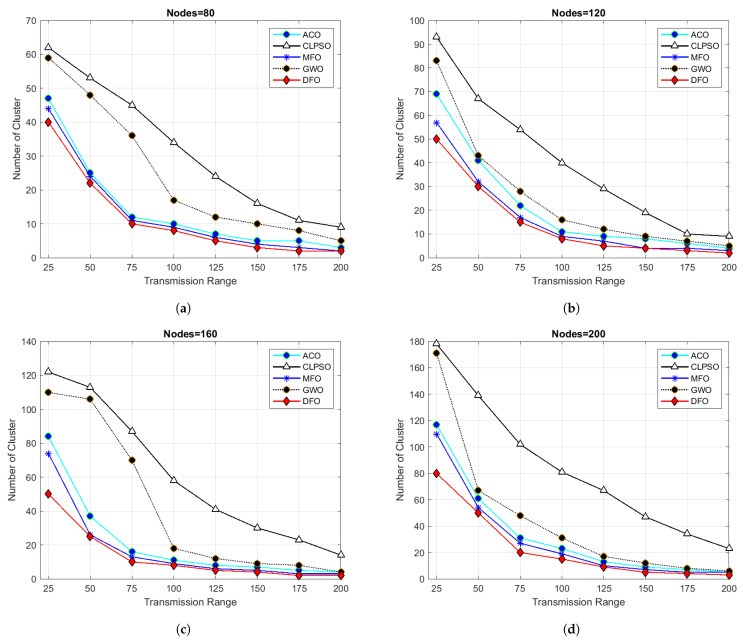
Grid size 500 m × 500 m and transmission range from 25 to 200.

**Figure 9:**
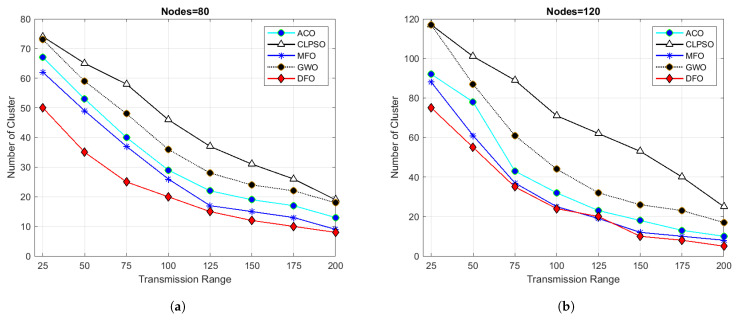

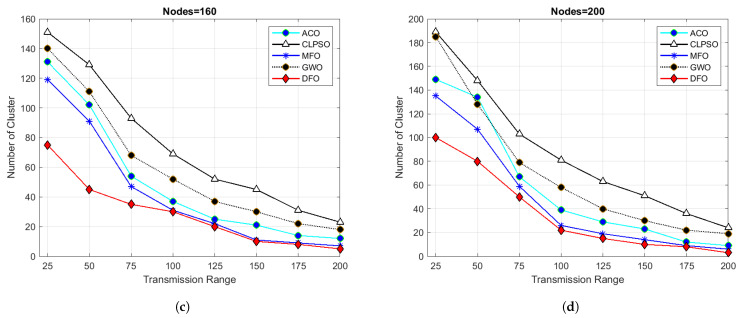
Grid size 1000 m × 1000 m and transmission range from 25 to 200.

**Figure 10:**
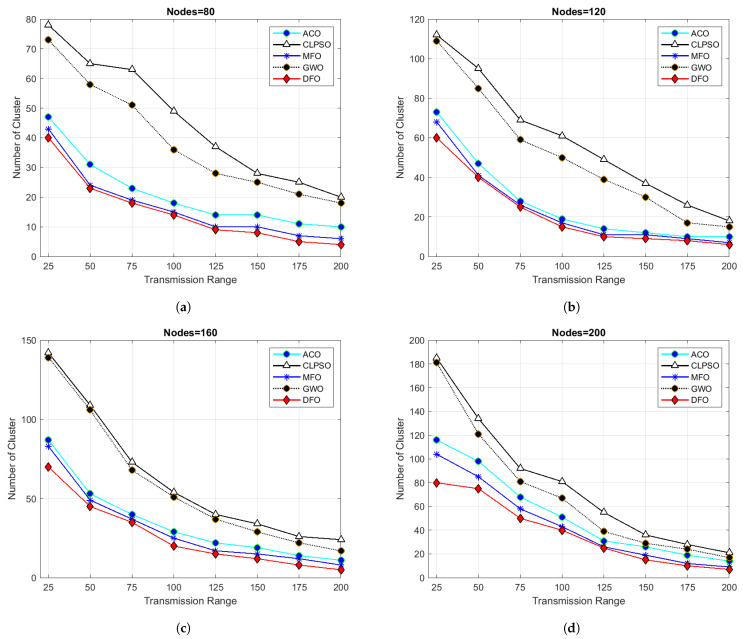
Grid size 1500 m × 1500 m and transmission range from 25 to 200.

**Figure 11:**
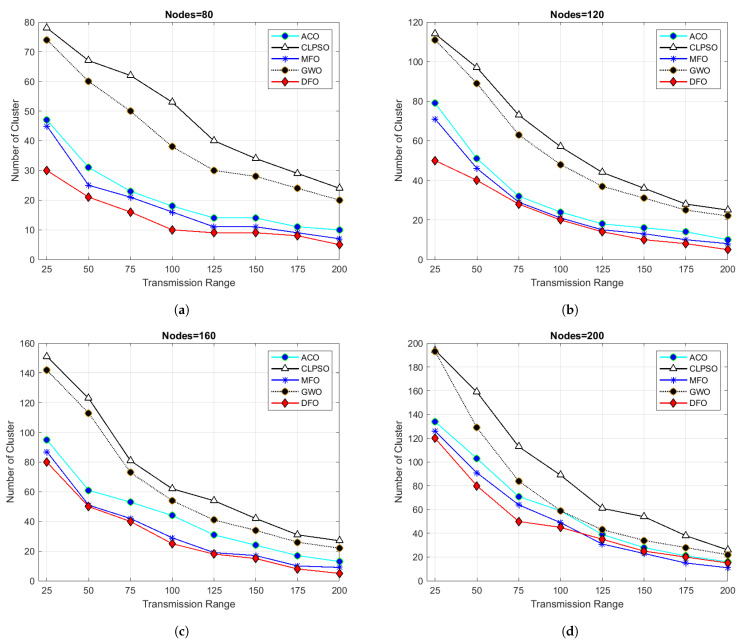
Grid size 2000 m × 2000 m and transmission range from 25 to 200.

For evaluating the results of all algorithms, 100 simulations were accomplished and results delineated in Figure 4, Figure 5, Figure 6, Figure 7, Figure 8, Figure 9, Figure 10 and Figure 11. Two fundamental parameters, number of nodes and transmission range, were assessed with distinctive values for checking the adequacy of ANC-UWSNs. Consequences demonstrate the suppleness and dominance of ANC-UWSNs over other algorithms.

In Figure 4, the transmission range of nodes is set from 50 m to 200 m with a fixed grid scale (500 m × 500 m) and nodes density is set from 20 to 200 nodes. Results demonstrate that even with varying transmission range DFO give good results than all other algorithms in comparison and show continuity of batter results in all scenarios, alhough MFO shows closest results to DFO in same scenarios. In Figure 4a, when the transmission range of each node was set upto 50 m in a fixed grid size of 500 m × 500 m and total number of nodes was taken from 20 to 200, then number of clusters formed by DFO was 30, and number of clusters formed by MFO, ACO, GWO and CLPSO respectively was 55, 60, 62 and 140. In Figure 4b, when transmission range was increased from 50 m to 100 m then number of clusters formed by DFO was 10 and number of clusters made by MFO, ACO, GWO and CLPSO was 19, 23, 31 and 81. Similar kinds of results are shown by Figure 4c,d transmission range was increased to 150 m and 200 m. It is evident from the results that, at the point when we increment the transmission range of nodes, the entire clusters created by all algorithms decreased and DFO create optimized number of clusters in given scenario as compared to other algorithms. Results show that the effects of a transmission range on the amount of clusters formed, with increased transmission range less number of clusters formed resulting in optimized routing. It is obvious from simulation results that increasing the node’s transmission range ultimately reduces the number of clusters formed, and DFO outperforms in all instances.

#### Grid Size VS Number of Nodes VS Number of Clusters

An additional test set was granted to verify the efficiency of the provided algorithm to make problems more tangible. Node density in these experiments ranged from 80 to 200 nodes; the transmission range was set from 25 m to 200 m in a fixed grid size of 500 m × 500 m.The experimental results are revealed in Figure 8.

In Figure 8a, a node density of 80 nodes was taken with transmission range of 25 m in fixed grid size of 500 m × 500 m. DFO created 40 clusters, while MFO generated 44 clusters, ACO 47, GWO 59 and CLPSO 61. When transmission range of each node was increased to 200 m then DFO generated only 2 clusters, MFO 3, ACO 5, GWO 6 and CLPSO 9 clusters. This is reliable with well-established reading and if the nodes transmission range is bigger, minimum clusters will be made. Results shown in figure show that DFO outperforms other algorithms even if transmission range varies from 25 to 200. It is obvious by seeing the results in Figure 8a–d having number of nodes 80, 120, 160 and 200 m, respectively, DFO outperforms other algorithms and creates a lower number of clusters in a given space, which helps in optimizing routing in UWSNs.

When we increase the grid size to up to 1 km, DFO outperforms all other algorithms. Figure 9 shows the outcomes of this setting. In this scenario, when density of nodes were fixed at 80 nodes then 8 clusters were created by DFO. In comparison, MFO produced nine clusters, ACO 14, GWO 18 and CLPSO produced 19 clusters. DFO evidently provided lesser number of clusters. When a node’s density was extended from 80 to 200, DFO generated four clusters while MFO created six clusters, ACO 9, GWO 20 and CLPSO generated 23 clusters. It results that with any number of nodes dragon fly algorithm generated small number of clusters which are very helpful in routing optimization problems. It can also be seen that the transmission range influenced the number of clusters generated. This is consistent with previous findings that if transmission range is smaller, more clusters are shaped, as there tend to be fewer nodes in a cluster. The nodes will also find closer nodes when the range is extended, which results in fewer clusters.

In order to further evaluate ANC-UWSNs efficiency, the calculations were tested by changing the transmission range of nodes while the grid size and number of nodes were retained at a fixed value, i.e., transmission range one km and node density fixed to 200 nodes. As shown in Figure 5, the results suggest that in the given case, DFO was a good solution and produced optimal results. Further simulations were performed while the grid size was increased to 1500 m. Figure 10 describes the consequences of this scenario. From Figure 10a, it could be verified that with a smaller transmission range of all algorithms, excluding the DFO, yielded similar findings, while the DFO yielded best possible results, viewing the algorithm’s effectiveness. It may also be evidenced that ANC-UWSNs efficiency also enhanced with the increasing number of nodes. For example, DFO created only seven clusters with density of nodes fixed to 200 nodes, whereas MFO made nine clusters, ACO 14, GWO 18 and CLPSO generated 22 clusters. DFO was assessed more with varying transmission range of 50–200 m, as with the previously mentioned settings, and the grid size was kept at 1500 m. Outcomes are shown in Figure 6. It is important to mention that there was a significant difference in output among DFO and other methods, when transmission range was fixed at 200 m. This demonstrates that the efficiency of DFO was enhanced with a greater transmission range and it created very few cluster numbers.

Results in Figure 11 show that DFO provides good results even with grid sizes of up to 2 km. In this scenario, when the transmission range was set to 100 m and 80 nodes were taken, then results demonstrated that GWO and CLPSO show poor results by generating 39 and 52 clusters, respectively. MFO and ACO comparatively give batter results by generating 17 and 19 clusters. DFO performs superbly by creating only ten clusters in the same scenario. When the transmission range was increased to 200 m, then DFO generated only six clusters and the closest one MFO generated nine clusters. Similar results can be seen even by changing different parameters such as transmission range, grid size and node density. It is proven from the results that DFO provides an optimal number of clusters in different experimentation. Hence, a DFO-based protocol (ANC-UWSN) is better for optimal routing in UWSNs than other algorithms. This creates an optimal number of clusters. Similar outcomes were observed when the transmission range was set to 50 m, 100 m, 150 m, and 200 m, with 200 nodes and a fixed grid size of 2 km^2^. By Figure 7 it is obviously demonstrated that the outcome of DFO was better than all other protocols in any specified scenario. By viewing the assessments, it is evident that ANC-UWSNs has performed significantly better than other techniques. It generated the lowest number of clusters needed to function in the specified grid size.

Energy efficiency is achieved by reducing the number of clusters in the network. The lesser the number of clusters, the easier it is to manage them. By having a lower number of clusters, few CHs are responsible to communicate with sink node when required. Therefore, the life of a sink node also increases. Hence, energy efficiency is indirectly achieved in our proposed methodology.

## 6. Conclusions

In this article, an adaptive node clustering protocol was proposed for UWSNs. The protocol uses a dragon fly optimizer for generating an optimal number of clusters in a given space. ANC-UWSNs uses an evolutionary-based algorithm which works repeatedly over a given space and generates an optimal number of clusters for packet routing. The cost of routing packets decreases as the number of clusters decreases in a given space. In this way, the overall routing cost of the network decreases and energy conservation of nodes decreases because only cluster head nodes participate in the routing of data packets. Altered simulations were performed for checking the efficiency of offered algorithm. The algorithm was tested with the variation of two main parameters, with the variation of node density and varying transmission range of nodes. The result of simulations shows that ANC-UWSNs is an efficient solution for routing in supposed networks. ANC-UWSNs was compared with other well-known evolutionary algorithms (MFO, GWO, ACO and CLPSO) and results show the dominance of DFO.

Future work can be carried out to investigate the affects of other critical parameters like the consumed power and residual energy which are also among the main constraints of underwater sensors.

## Figures and Tables

**Table 1:** Simulation parameters for algorithms.

Algorithms	Inertia	Evaporation Rate	*C_min_* & *C_max_*	Grid Size	Node Density	Mobility	Transmission Range	Distance between Nodes	*w*_1_ & *w*_2_
ACO [51]	-	0.5	2	500 × 2000 m^2^	20–200	Fixed	25–200 m	±5	0.5
MFO [52]	0.90	-	2	500 × 2000 m^2^	20–200	Fixed	25–200 m	±5	0.5
GWO [53]	0.694	-	2	500 × 2000 m^2^	20–200	Fixed	25–200 m	±5	0.5
CLPSO [54]	0.694	-	2	500 × 2000 m^2^	20–200	Fixed	25–200 m	±5	0.5
DFO [47]	0.694	-	2	500 × 2000 m^2^	20–200	Fixed	25–200 m	±5	0.5

## Abbreviations

The following abbreviations are used in this manuscript:

UWSNs	Underwater sensor networks
ANC-UWSNs	Adaptive node clustering technique for underwater sensor networks
ACO	Ant colony optimizer
GWO	Gray wolf optimizer
MFO	Moth flame optimizer
CLPSO	Comprehensive learning particle swarm optimizer
DFO	dragonfly optimizer
UOC	Underwater optical communication
FANET	Flying ad-hoc network
CH	Cluster head
WSNs	Wireless sensor networks
UAC	Underwater acoustic communication
VANET	Vehicular ad-hoc network
